# CoffeeProt: an online tool for correlation and functional enrichment of systems genetics data

**DOI:** 10.1093/nar/gkab352

**Published:** 2021-05-12

**Authors:** Jeffrey Molendijk, Marcus M Seldin, Benjamin L Parker

**Affiliations:** Department of Anatomy and Physiology, University of Melbourne, Melbourne, VIC 3010, Australia; Department of Biological Chemistry and Center for Epigenetics and Metabolism, University of California, Irvine, CA 92697, USA; Department of Anatomy and Physiology, University of Melbourne, Melbourne, VIC 3010, Australia

## Abstract

The integration of genomics, transcriptomics, proteomics and phenotypic traits across genetically diverse populations is a powerful approach to discover novel biological regulators. The increasing volume of complex data require new and easy-to-use tools accessible to a variety of scientists for the discovery and visualization of functionally relevant associations. To meet this requirement, we developed *CoffeeProt*, an open-source tool that analyses genetic variants associated to protein networks, other omics datatypes and phenotypic traits. *CoffeeProt* uses transcriptomics or proteomics data to perform correlation network analyses and annotates results with protein-protein interactions, subcellular localisations and drug associations. It then integrates genetic variants associated with gene expression (eQTLs) or protein abundance (pQTLs) and includes predictions of the potential consequences of variants on gene function. Finally, genetic variants are co-mapped to molecular or phenotypic traits either provided by the user or retrieved directly from publicly available GWAS results. We demonstrate its utility with the analysis of mouse and human population data enabling the rapid identification of genetic variants associated with druggable proteins and clinical traits. We expect that *CoffeeProt* will serve the systems genetics and basic science research communities, leading to the discovery of novel biologically relevant associations. *CoffeeProt* is available at www.coffeeprot.com.

## Graphical Abstract

**Graphical Abstract:**
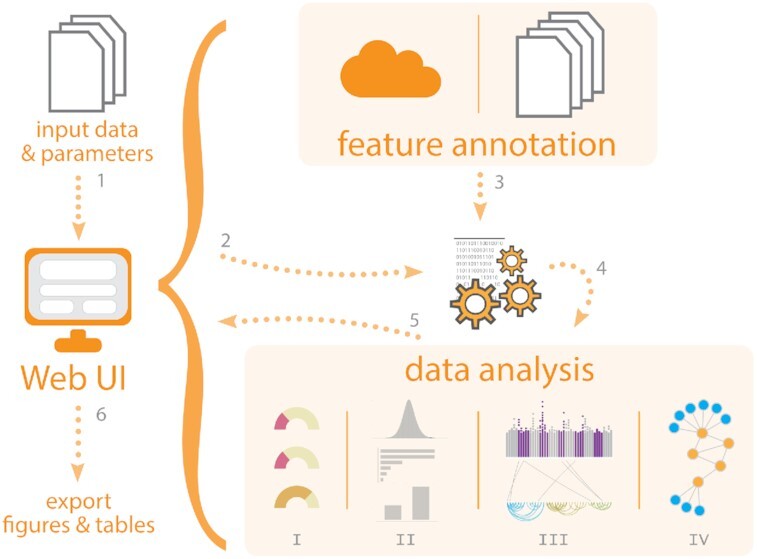
CoffeeProt: An online tool for correlation and functional enrichment of systems genetics data.

## INTRODUCTION

The field of genetics has realized significant progress in the discovery of phenotype-associated genetic variation in recent years (1). As of February 2021, over 247,000 genetic associations have been extracted from more than 11,600 genome-wide associations studies (GWAS), summarised in the GWAS Catalog (2). This success is attributable to technological advances, access to increasing amounts of genetic and phenotypic data, and the continuous development of novel analytical tools. The functional relevance of phenotype–genotype associations in diverse populations and environments is a rapidly evolving and challenging area in deciphering molecular mechanisms of complex health and disease traits. Systems genetics is an approach in which intermediate molecular phenotypes are examined in relation to genetic variation to improve our understanding of complex traits and common diseases (3). Evidently, the integration of different biological layers has distinct advantages over the analysis of a single biological layer as the flow of information can be modelled to identify and prioritize core regulators for functional validation (4). Linking genetic loci to complex traits via association analysis can be better understood through linking quantitative trait loci (QTL) across biological layers. Such examples could include proteins (pQTL), transcripts (eQTL) and other molecular traits (e.g. molQTL) (5). However, a significant challenge of these studies is that the number of associations can be very large, requiring complex integrations, filtering and data visualizations to interpret biologically relevant interactions and discover potential new causal relationships for subsequent validation. Collated lists of computational resources used in the field of systems genetics are available (6), such as Mergeomics (7), WGCNA (8), MEGENA (9), MOFA (10), intermediate (11) and ARACNE (12). These tools are used to identify key drivers in biological pathways, constructing gene co-expression networks, performing mediation analysis and inferring relationships between quantitative measures however, usage of such resources requires knowledge of computational languages such as Python or R. ProGem is a recent tool developed by Stacey *et al.* to identify and prioritize causal genes at molecular QTLs (13). This powerful framework leverages positional and QTL data combined with pathway analysis to prioritize possible genes underlying the biological mechanisms. Additionally, LipidGenie (14), GeneNetwork (15) and Systems-Genetics.org (GeneBridge & multispecies expression compendia) (16) are resources that enable the browsing of QTLs or phenotype associations from various genetic cohorts, but do not allow the analysis of user-generated datasets. Some limitations of all the tools mentioned above are that they either require advanced experience in computational analysis, are not available online or they do not have linked visualization features. This means they may not be accessible to a broad range of scientists and the time required to learn these computational protocols may be prohibitive. Furthermore, many of these tools are not focused on the inclusion of proteomic data with key annotations such as protein-protein interactions or subcellular localisation. The inclusion of this data offers exciting opportunities to further investigate the mechanisms of genotype and trait associations. For example, the integration of pQTLs with protein-protein interaction networks has the capacity to define genetic regulation of protein complexes (11) or the inclusion of subcellular localisation and molQTLs can identify compartmentalized associations (17). However, the annotation of large volumes of systems genetic data to identify, visualize and prioritize functional assessment of key regulators remains a daunting task.

**Figure 1.**
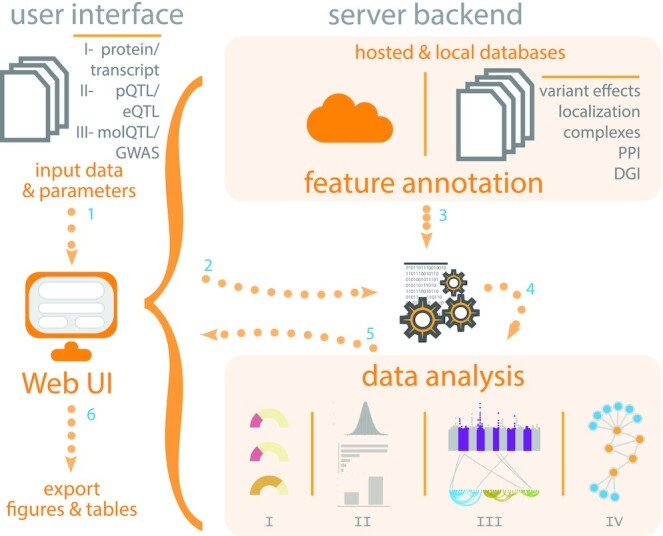
CoffeeProt workflow. The *CoffeeProt* workflow starts with users accessing the *CoffeeProt* web user interface to upload datafiles and specify analysis parameters (1). The user interface and server backend running R are connected using the shiny R package (2). Feature annotations are performed based on local databases included in *CoffeeProt* as well as remotely hosted databases on the Melbourne Research Cloud (3). User data is analysed to perform summary statistics (I), correlation (II), interaction (III) and network (IV) analyses (4). The results are displayed in the web interface for result interpretation by the user (5). Finally, all individual tables and plots can be exported (6). (QTL: quantitative trait loci; PPI: protein–protein interaction; DGI: drug–gene interaction).

Here, we present *CoffeeProt*, an easy-to-use online tool to enable the integrated analysis of transcriptomics and/or proteomics data with combined genetic/molecular phenotypic associations and functional annotations followed by higher-order visualizations (Figure 1). *CoffeeProt* differs from existing tools by performing co-expression analyses and annotates findings in the context of protein-protein interactions and subcellular localisations and seamlessly integrates QTL data with interactive networks and visualizations all in the one online platform. Importantly, usage of *CoffeeProt* requires no bioinformatics or coding experience. Resources including the GWAS Catalog (2), the Drug Gene Interaction Database (DGIdb) (18) and Ensembl variant effect (19) annotations are integrated in the application, allowing for easy access to publicly available datasets and variant annotations. By allowing users to upload a variety of data types, combined with the annotation of several existing resources with novel visualisation in a single workflow, *CoffeeProt* significantly reduces the time users would spend processing and integrating multi-omic data. Ultimately, this tool greatly enhances the ability to gain biological insights from systems genetics data. We believe that our interactive workflow offers advantages in the inspection of biological associations and the prioritization of candidates, as the conclusions drawn from a biological network rely greatly on its annotations and the level of displayed genetic information. Ultimately, *CoffeeProt* enables the rapid identification and prioritization of functionally relevant targets for follow up studies. *CoffeeProt* is freely accessible through an online user interface at www.coffeeprot.com, allowing analyses to be performed without programming knowledge or software installation.

## MATERIALS AND METHODS

### Data input & processing

The first step of the *CoffeeProt* workflow involves the user to upload input data: (i) transcriptomics or proteomics data matrix, (ii) eQTL or pQTL data, (iii) and GWAS or molQTL datasets using the web interface (Figure 1). Example input files are available to download from the *CoffeeProt* welcome page. Datasets can be uploaded as comma separated .csv files, tab separated .txt files or Excel files in .xls or .xlsx formats. *CoffeeProt* automatically detects the file format and validates the dataset prior to data processing. Users can upload only (i) and/or (ii) datasets and skip (iii) GWAS/molQTL associations if they choose.

Transcriptomics/proteomics data must be uploaded as a matrix in which the first column contains identifiers, and the remaining columns contain quantitative measurements. The accepted identifiers are gene symbols, Uniprot identifiers and Ensembl gene identifiers. *CoffeeProt* automatically detects the identifiers and converts them to gene names during the processing steps since these identifiers are required for several downstream analyses. Missing values in the data can be addressed by imputation or by removing proteins of which the number of missing values exceeds the cut-off defined by the user. Pair-wise protein-protein correlation (i.e. co-expression or co-regulation analyses) can be performed using either the Pearson correlation coefficient (PCC), Spearman's rank correlation coefficient or biweight midcorrelation (bicor) as implemented in the WGCNA R package (20). Following the correlation analysis and correction for multiple hypothesis testing with either Benjamini-Hochberg or Bonferroni, a list of protein pairs and their co-regulation metrics is created including the *P*-value, *q*-value and correlation coefficient.

For the e/pQTL data upload the list should contain separate columns containing (i) RefSeq identifiers (rsIDs), (ii) SNP location, (iii) SNP chromosome, (iv) gene names, (v) gene start location, (vi) gene end location, (vii) gene chromosome, (viii) a measure of significance and (ix) a proxy or grouping column. Regarding the SNP and gene location columns, measures such as physical position (bp) or genetic distance (cM) are accepted. Non-cumulative measures (e.g. a measure that starts at zero for each chromosome) will be converted to a cumulative relative SNP location to allow the generation of Manhattan plots. The measure of significance column should contain either *P*-values or logarithm of the odds ratio (LOD) scores. Filtering of the e/pQTL dataset is allowed using a single measure of significance, or separate cut-offs for each QTL proxy. A proxy can be specified to allow separate cut-offs when the dataset contain both SNPs located near the gene it affects (local or *cis*-e/pQTLs) or SNPs that affect genes located in other regions of the genome (distant or *trans*-e/pQTLs). Due to the large file sizes common in QTL files, it is recommended to filter QTL data prior to uploading to *CoffeeProt*, to reduce the time required to upload data.

The third type of data upload is the association to phenotypic or molecular traits (GWAS or molQTLs). These could include associations such as metabolite-QTLs (metQTLs), lipid-QTLs (lQTLs), or others generated by the user. *CoffeeProt* enables easy access to publicly available data from the GWAS Catalog (2) for instances where the user only has access to proteomics and pQTL data. The required data format is like the e/pQTL upload columns mentioned above however, instead of gene/protein names, a molecule or phenotypic trait is listed as associated to the genetic variants. For the molQTL/GWAS data upload the matrix should contain separate columns containing (i) RefSeq identifiers (rsIDs), (ii) phenotypic trait(s), (iii) SNP location, (iv) SNP chromosome, (v) a measure of significance and (vi) a grouping column. The use of molQTL/GWAS in *CoffeeProt* allows for the discovery of SNP-protein associations that share associations with molecular or clinical phenotypes via a co-mapping SNP. The molQTL/GWAS tab in *CoffeeProt* contains a table listing all published studies in the GWAS Catalog, including the journal article title, publication date and traits investigated. A user can simply select a study of interest and click the download button to retrieve a list of variants associated to phenotypes that are directly usable as input molQTL data in *CoffeeProt*. The GWAS Catalog representational state transfer (REST) application programming interface (API) is accessed through the gwasrapidd R package (21). First all datasets related to the user selected study are retrieved using the get_associations function, followed by variant annotation using the get_variants function. Finally, the data table is trimmed to retain the six columns required for further analysis in *CoffeeProt*.

### Annotation

Imported proteins are annotated using subcellular localisations from the Cell Atlas as determined using immunofluorescence (22). To address the varying specificity of the localisations in the database, we also added the ancestor localisations from QuickGO (23) for overly specific annotations. For example, a protein present in a nuclear speck is also considered to be present in the nucleoplasm and the nucleus. This step is essential in determining whether two correlated proteins are located in the same organelle. Furthermore, drug interactions from the Drug Gene Interaction Database (DGIdb) are searched and used in several visualizations (18). All protein-protein correlation pairs are searched against the STRING database (24), as well as the CORUM (25) and BioPlex 3.0 (https://bioplex.hms.harvard.edu/interactions.php) (26) protein–protein interaction databases to detect previously reported associations. For the interactions in the BioPlex 3.0 database we only considered the top 10th percentile (11817/118162) as correlated protein–protein interactions based on a previous publication by Huttlin *et al.* showing significant overlap between CORUM protein-protein interactions and the top BioPlex interactions (27). A PostgreSQL database (v 11.9) hosted on the Nectar Research Cloud is used for the annotation of RefSeq identifiers in the e/pQTL datasets. This database contains both rsIDs and variant effects defined by the Sequence Ontology (28) which were retrieved from the latest Ensembl variation database (v100) (ftp://ftp.ensembl.org/pub/current_variation/vcf/) (19). Additionally, the rsIDs are assigned variant consequence impact ratings as used by variant annotation tools such as snpEff (29). Impact ratings refer to the disruptive effects the variant has on the functioning or effectiveness of a protein. High-impact variants are likely to cause protein truncation, loss of function or the triggering of nonsense mediated decay. A moderate-impact variant consequence is non-disruptive but may cause changes in protein activity or function such as an inframe insertion or deletion. A low-impact variant in unlikely to cause a change in protein behaviour such as a synonymous variant. Finally, modifier-impact variant consequences affect non-coding areas are also annotated and these may influence the expression of the protein such as variants located in transcription factor binding sites or other regulatory elements (29).

### Analysis and visualization

*CoffeeProt* allows the customization of figures with user-selected cut-offs and the interactive selection of target genes/proteins, complexes, and phenotypes or molecular traits. These data are integrated via network visualizations to ultimately understand how genetic variants are associated to protein complexes and different biological layers or phenotypes. *CoffeeProt* initially displays several plots related to the filtering and annotations specific to each of the uploaded datasets. Examples of these quality control plots are shown in Supplementary Figures S1 and S2. For transcriptomics/proteomics datasets, multiple gauge charts are produced to highlight the number of proteins filtered by the missing value cut-off, and the number of proteins annotated by protein localisation or annotated with known drug interactions. Similarly, for QTL datasets multiple donut charts highlight the distribution of QTLs annotated by the proxy, grouping column, variant effects and variant impact ratings.

Following pair-wise correlation analysis, a summary tab contains visualizations related to the protein-protein correlation analysis. Histograms show the distribution of correlation coefficients or corresponding regression *q*-values, highlighting the proportion that are associated based on user-defined cut-offs. Additionally, the number of correlated partners per protein is shown to highlight the proportion of highly connected proteins in the data set. To assess the database overlap of co-regulation data, chi-squared tests of independence are performed to determine the relationship between shared database presence and correlation coefficients of protein-protein pairs. Co-regulated proteins are expected to be enriched in the same cellular location and the same protein complexes as previously shown by Kustatscher *et al.* (30). The interactive nature of *CoffeeProt* allows users to adjust correlation coefficient or *q*-value cut-offs to ensure functionally related protein pairs are significantly enriched. Sensitivity analyses can be performed using *CoffeeProt* to assess the effects of these input parameters on the fraction of protein pairs present in the reference databases. Over 70 combinations of correlation coefficient and *q*-value cut-offs are tested for the different databases and visualized (e.g. Supplementary Figure S3)

*CoffeeProt* has several functionalities to explore and visualise interactions between genetic variants and the various biological layers. First, a Manhattan plot can be created using the uploaded e/pQTL data, highlighting the loci associated with the abundances of specific transcripts or proteins. Next, we introduced a novel visualisation to display co-regulated protein networks linked to the Manhattan plot to understand how genetic variants may regulate protein complexes. Finally, several interactive network plots can be created using one or multiple biological layers with nodes containing SNPs, transcripts, proteins or molecular/phenotypic traits. These network plots can be customised by filtering interactions previously reported in the CORUM or BioPlex 3.0 databases or proteins present in an organelle of interest, by annotating the network with drug interactions, or by dynamically modulating significance thresholds. Furthermore, edges of the networks can be annotated with proxies (i.e *cis*- versus *trans*-associations) or variant effect predictions. The networks can be modified to show all the associations of a single gene, a co-regulated complex or all the associations to a molecular/phenotypic trait of interest. To reduce the complexity of the network plots which are products of genetic linkage, it is possible to summarize the large number of individual SNPs into chromosome nodes or linkage disequilibrium (LD) block nodes. To enable the latter option, a three-column LD block file containing the chromosome, start location and end location of the LD blocks should be uploaded along with the eQTL/pQTL data. The chromosome location format in the LD block should match the format in the QTL data files. A significant feature of *CoffeeProt* is the identification and visualization of associations between transcripts/proteins and molecular or phenotypic traits based on shared co-mapped SNP rsIDs. This is important because it allows rapid identification of potential causative genes or proteins which underlie associations between significant SNPs and phenotypic or molecular traits for functional validation.

### Reporting

After completing the analysis workflow, it is possible to download all figures and tables produced by the user. These processed and annotated datasets required to produce all images can be exported as .csv files for further analysis outside of *CoffeeProt*. All plots created in the tool can be directly exported in various formats, including vector-based images in .svg and .pdf formats and in various dimensions. Alternatively, zip compressed folders containing all plots or tables can be downloaded. The interactive network plots can be downloaded as .html files.

### Web server implementation

*CoffeeProt* was developed using the R programming language for the backend and relies on the shiny package for the web server front-end in addition to HTML, CSS and JavaScript. Several tidyverse packages are used for data wrangling and data visualization. Furthermore, *CoffeeProt* relies on the WGCNA package for the Pearson's, Spearman's and bicor correlation analyses (8). Circos and interactive network plots are created using the circlize (31) and networkD3 R packages respectively. The GWAS Catalog is accessed using the gwasrapidd package (21). *CoffeeProt* is deployed on the Nectar Research Cloud and Melbourne Research Cloud, utilizing hypervisors built on AMD EPYC 2 (base CPU clock speed 2.0 GHz, burst clock speed 3.35 GHz) and running Ubuntu 18.04.

### Privacy and security

The uploaded data is only available to be analysed if the user is connected to the *CoffeeProt* server. No user data is retained after the analysis session has been terminated. *CoffeeProt* does not require raw genomics or proteomics data and the user can de-identify sample information. Secure HTTPS connections can be used to transfer data to, and from the *CoffeeProt* servers.

## RESULTS

### Case study: An integrative systems genetic analysis of mammalian lipid metabolism

**Figure 2.**
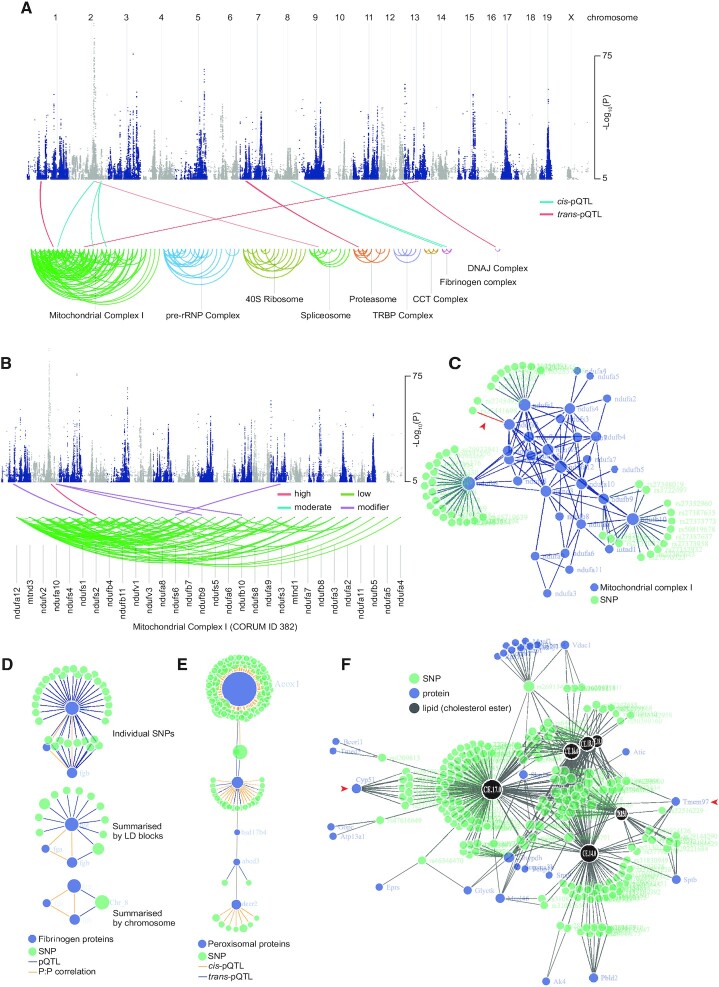
Visualisation of associations between SNPs and the abundance of protein co-regulated networks or lipids. (**A**) QTL-protein associations of all correlated CORUM protein networks with associations coloured by *cis*- or *trans*-proxy. (**B**) SNPs associated to Mitochondrial complex I subunits with associations coloured by variant impact. (**C**) Network plot revealing SNPs associated to Mitochondrial complex I subunits. (**D**) Network of SNPs associated with fibrinogen proteins shown as individual SNPs, summarized into LD blocks or chromosomes. (**E**) Network of proteins located in peroxisomes (vesicles), according to Human Protein Atlas annotations. (**F**) Phenotype bait-network showing co-mapping SNPs to proteins and cholesterol ester (CE) lipid species in which CE 14:0. CE 15:0. CE 16:0, CE 17:0. CE 18:0 and CE 18:1 were selected as targets.

To illustrate the utilities of the *CoffeeProt* tool, we analysed data previously published by Parker *et al.* (17). Here, liver proteomic and lipidomic analysis was performed across >100 genetically diverse inbred mouse strains (>300 individual mice) and integrated with genomic data to identify pQTLs and lipid-QTLs (lQTLs). A detailed description of the data and quality control analysis performed in *CoffeeProt* is presented in the Supplementary Material and Supplementary Figure S1. To visualize the associations between genetic variation and co-regulated protein networks, *CoffeeProt* produces SNP-protein plots. Figure 2A displays a Manhattan plot created using pQTL data and loci are linked by edges to co-regulated networks depicted as arc diagrams. The edge starts at the position of the SNP underneath the Manhattan plot and ends on the associated protein underneath. The protein is linked to other correlated proteins via arc diagrams which are grouped according to their CORUM protein complexes. In the protein arc diagram, complexes are first arranged by size (largest to smallest), and individual proteins are ordered by the number of protein-protein correlations (most to least). The lines linking the loci to the co-regulated networks are further annotated with the *cis-/trans*-proxies based on the genomic proximity of each SNP to its associated protein. In Figure 2B, Mitochondrial Complex I (CORUM ID 382) has been selected to investigate variants associated to precise subunits which have now been annotated with impact ratings. In this example, a high-impact *trans*-pQTL associated to NDUFS2 has been identified and analysis of the annotated pQTL table identifies a *trans*-acting variant in a predicted splicing acceptor site (rs27441698) within the *Sptbn5* gene.

We next demonstrate *CoffeeProt's* ability to generate multi-omic visualizations via interactive networks. Users can select various co-regulated networks based on the CORUM or BioPlex databases and integrate associated genetic variants as nodes with links annotated by proxy, variant effects or impact ratings. Figure 2C displays an alternative network view of the pQTLs associated to Mitochondrial Complex I with the example high-impact association mentioned above shown in red and indicated by a red arrowhead. A further co-regulated network highlighting fibrinogen proteins FGA, FGB and FGG is shown in Figure 2D. Here, several co-mapping individual SNPs are presented in the top network with 41 *trans*-pQTLs located on chromosome 8. These SNPs can be grouped and summarised based on LD block nodes shown in the middle network or based on chromosome nodes shown in the bottom network. Interestingly, out of the 41 individual SNPs, 36 are located within known LD blocks, with a single LD block on chromosome 8 connecting the FGB and FGG proteins. Networks can also be created showing only proteins located in an organelle of interest (Figure 2D). Here, the peroxisomal proteins ACOX1-CAT-HSD17B4-ABCD3-DECR2 are connected through protein-protein interactions and through shared genetic mutations. *CoffeeProt* also allows users to perform a molecular or phenotypic-centric network analysis to investigate potential upstream protein and genetic regulators. Figure 2F highlights co-mapping pQTLs and lQTLs to hepatic cholesterol ester abundance. Two previously characterized regulators of cholesterol metabolism are highlighted with red arrows including CYP51, a monooxygenase catalysing the first step in the conversion of lanosterol into cholesterol (32), and TMEM97, a lysosomal protein associated with the regulation of low-density lipoproteins (LDLs) and cholesterol ester metabolism (33). These examples demonstrate *CoffeeProt's* ability to identify previously reported regulators of lipid metabolism but also highlight many more uncharacterized associations for future functional validation.

### Case study: Genomic atlas of the human plasma proteome

**Figure 3.**
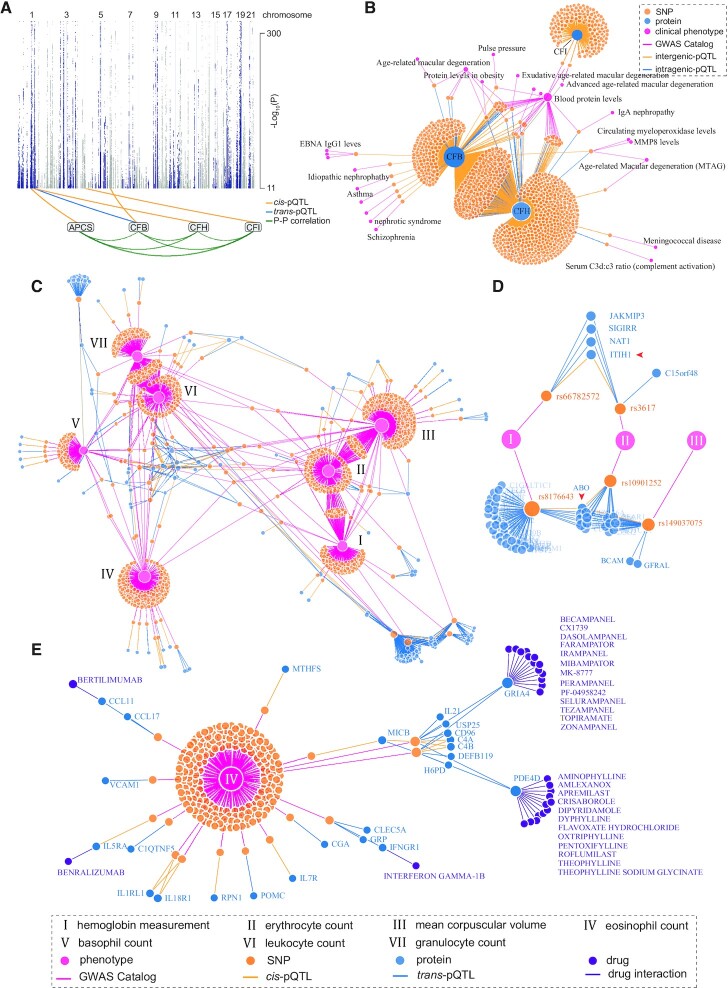
Integration of co-regulation networks, pQTL and GWAS in human plasma samples. (**A**) A SNP-protein association plot highlights *cis-* and *trans-*pQTLs associated with a co-regulated protein network of complement factors. (**B**) The CFB-CFH-CFI network reveals shared SNPs between the proteins and clinical phenotypes from the GWAS Catalog. (**C**) Phenotype network of blood measurements and cell counts associated to plasma pQTLs. (**D**) Proteins associated with SNPs located on or near ABO and ITIH1. The red arrowheads indicate proteins with *cis*-pQTL associations. (**E**) Phenotype bait-network of the eosinophil count phenotype associated with pQTLs. Drug interactions from the DGIdb were integrated with proteins in the network.

As a second case study we used *CoffeeProt* to analyse genetic associations of the human plasma proteome from the INTERVAL study published by Sun *et al.* (34). Here, plasma proteomic analysis was performed across >3,300 healthy participants and integrated with genomic data via pQTL analysis. A detailed description of the data and quality control analysis performed in *CoffeeProt* is presented in the Supplementary Material and Supplementary Figure S2. We initially investigated *cis*-pQTL and focused on intragenic SNPs (i.e within introns or exons) given their greater potential to impact protein function and investigated their associations to the abundance of co-regulated protein networks. Figure 3A displays a SNP-protein plot with pQTLs associated to co-regulated protease complement factors CFB, CFH, CFI and APCS. *CoffeeProt* identified previously characterized high-impact SNPs including rs4151667 and rs641153, resulting in non-synonymous L9H and R32Q variants of CFB, respectively (35,36). A total of 57 *cis*-acting variants associated to CFH were also associated to CFB in *trans*- such as rs371972000 which is located <5kB to the transcriptional start of CFH. Given the abundances of these two proteins are correlated, these data suggest genetic regulation of CFH *in cis* subsequently regulates the abundance of CFB which warrants further functional validation. Integrating co-mapping SNPs between protein abundance and phenotypic traits (GWAS Catalog, all associations file from 2020-10-07, v1.0.2, *P* < 1e^–11^) (Figure 3B) highlights several well characterised intragenic co-mapping SNPs associated to CFH and CFB, and age-related macular degeneration (37) including several other less characterised phenotypic associations such as potential links between CFB, CFH and obesity.

We next used *CoffeeProt* to identify co-mapping associations between plasma pQTLs and 36 blood cell traits that were directly downloaded from the GWAS Catalog in *CoffeeProt* (38). Figure 3C displays associations to various blood cell counts and other clinical measures including mean corpuscular volume (MCV) and hemoglobin content. This interactive network provides an exciting glimpse into the complex genetic regulation of human blood and plasma proteome revealing several known and novel associations. Red blood cell associations are clustered on the right (I–III) while other blood cells (granulocyte, leukocyte, basophil, eosinophil) are clustered on the left (IV–VII). Figure 3D highlights previously characterized *cis*-acting SNPs in the ABO locus including rs149037075 and rs10901252 which *CoffeeProt* has annotated as 3 prime UTR variants, and rs8176643, annotated as an intron variant. The latter was validated in a meta-analysis of SNPs in the ABO locus and their associations with red blood cell traits (39), and agrees with a study by Emilsson *et al.* who identified over 40 proteins via *trans*-pQTLs associated with variants located on the *ABO* gene, which were also associated with cardiovascular disease and hemostasis (40). The traits ‘hemoglobin measurement’ and ‘erythrocyte count’ are both associated with two separate SNPs (rs66782572 and rs3617) which are *cis*-pQTLs for the protein ITIH1 (Figure 3D). This gene is a known marker for high-altitude adaptation and is differentially regulated in response to low-oxygen levels, but a causal role of this protein in red blood cell biology is unknown and warrants further investigation (41,42). We next expanded our analysis focusing on genetic and protein factors associated to eosinophil counts with the aim to identify potential drug interactions (Figure 3E). The variant rs1695315 was associated to eosinophil counts and is a *cis*-pQTL for IL5RA which was annotated as a target of the monoclonal antibody Benralizumab approved for the treatment of severe eosinophilic asthma and eosinophilic oesophagitis. *CoffeeProt* also identified rs2228467 as a *trans-*pQTL associated to eosinophil counts and Eotaxin (CCL11), a chemokine targeted by the monoclonal antibody Bertilimumab approved for the treatment of bullous pemphigoid in patients with eosinophilia. Importantly, both variants have recently been identified as causal for eosinophil counts using mendelian randomization (MR) analysis (43). While a recent phenome-wide MR study using >1000 plasma proteins included several bloody cell phenotypes (44), eosinophil counts were not included in the analysis and therefore further experiments are required to investigate potential causal roles of the identified proteins. Taken together, these examples highlight the power of *CoffeeProt* to rapidly integrate, visualize and interrogate multiple associations to complex human traits which includes an overview of known drug interactions but also highlights other potential druggable targets to potentially modulate eosinophil biology.

## SUMMARY

We present *CoffeeProt*, a novel online tool for the correlation and functional enrichment of proteome-wide systems genetics. *CoffeeProt* is flexible and accepts a variety of datatypes, starting with transcriptomics or proteomics data from population studies. The workflow significantly reduces the time users would otherwise spend on pre-processing, annotating, analysing and/or visualizing of their systems genetics data. The use of a dedicated database for SNP variant effects has allowed the rapid annotation of QTLs, enabling easy prioritization of associations based on predicted variant impacts. By allowing integrative analyses to be performed on co-regulated network analysis, e/pQTL, molQTL and phenotypic data, users can visualize and discover interactions and associations which may otherwise have been missed. Furthermore, mapping *cis*- and *trans*-e/pQTL data onto protein-protein co-regulated networks offers several advantages to investigate the genetic effects on protein complexes and potentially limit false positive associations by observing co-mapping SNPs. As shown in two case studies, *CoffeeProt* allows for the integration of multiple -omics datasets for the discovery of biologically relevant associations. We aim to support and continually improve *CoffeeProt* over the coming years based on user feedback and the changing bioinformatics requirements in the systems genetics field.

## DATA AVAILABILITY

*CoffeeProt* is accessible as a website (www.coffeeprot.com) and the source code is publicly available (https://github.com/JeffreyMolendijk/CoffeeProt). No login is required to use *CoffeeProt* and detailed documentation is provided on the website. The Parker HMDP data files used as case study 1 are included as demo files available to download through the *CoffeeProt* website. RefSeq identifiers and variant effects defined by the Sequence Ontology (28) which were retrieved from the latest Ensembl variation database (v100) (ftp://ftp.ensembl.org/pub/current_variation/vcf/). Subcellular protein localisation information was downloaded from the Human Protein Atlas (22) (https://www.proteinatlas.org/download/subcellular_location.tsv.zip). The Drug Gene Interaction Database (18) (October 2020 version) was downloaded from (https://www.dgidb.org/downloads). GENCODE human release 19 (CRCh37.p13) was used to retrieve genomic locations for the pQTL data in the second case study. The CORUM database was downloaded from http://mips.helmholtz-muenchen.de/corum/#download. BioPlex 3.0 was downloaded from https://bioplex.hms.harvard.edu/interactions.php. The data used in case study 2 (Sun *et al.*) are available through the European Genotype Archive (accession number EGAS00001002555).

## Supplementary Material

gkab352_Supplemental_File
