# Supplementary material for: CoffeeProt: an online tool for correlation and functional enrichment of systems genetics data
Source: Nucleic Acids Res. 2021 May 12;49(W1):W104–13. doi: 10.1093/nar/gkab352 (PMC8262721; doi:10.1093/nar/gkab352)
Supplement: gkab352_Supplemental_File [file gkab352_supplemental_file.pdf]

CoffeeProt: An online tool for correlation and functional enrichment of systems genetics data

Supplementary Material

Jeffrey Molendijk[1], Marcus M. Seldin[2] & Benjamin L. Parker[1*]

[1] Department of Physiology, University of Melbourne, Melbourne, VIC 3010, Australia
[2] Department of Biological Chemistry and Center for Epigenetics and Metabolism, University of California, Irvine, CA 92697, USA

* To whom correspondence should be addressed. Tel: +61401 758 489; Email: ben.parker@unimelb.edu.au

Case study data descriptions

CASE STUDY: An integrative systems genetic analysis of mammalian lipid metabolism

In this study, mass spectrometry-based liver proteomic and lipidomic data were measured in the Hybrid Mouse Diversity Panel (HMDP) and integrated with genomic data via QTL analysis to identify genetic variants associated to proteins and lipids (1). For this case study we downloaded the supplemental information (https://www.nature.com/articles/s41586-019-0984-y#Sec34) and simply adapted the tables to be compatible with the *CoffeeProt* tool. The proteomics data were filtered to exclude proteins with more than 20% missing values amongst the samples, leaving a dataset containing 2,253 proteins in 306 mouse liver samples (**Supplementary Figure 1A**). Among these proteins, 1,663 (74%) were annotated by protein localization as determined using immunofluorescent staining (**Supplementary Figure 1B**) and 646 (29%) were detected in the Drug-Gene Interaction Database (**Supplementary Figure 1C**).

Protein correlation summary

We performed a protein-protein correlation analysis to obtain biweight midcorrelation (bicor) coefficients, followed by the Benjamini-Hochberg procedure to obtain adjusted p-values (q-values). Even a relatively small proteomics dataset will lead to several million unique protein pairs, indicating the necessity to differentiate between truly associated protein pairs and sporadic correlations. Here we considered proteins with a bicor coefficient greater than 0.5 and q-value < 0.05 to be correlated. This correlation coefficient cut-off led to 0.15% ($3.86e^3$ out of $2.53e^6$) of the protein-pairs to be defined as correlated (**Supplementary Figure 1D-E**). Using these cut-offs, most proteins have no or a small number (1-5) of co-regulation partners (**Supplementary Figure 1F**). A smaller group of proteins has many co-regulation partners, indicating proteins that are likely central in one or multiple protein complexes.

Protein correlation database enrichment

Analyzing the fraction of previously reported protein pairs in the correlation matrix based on the CORUM and BioPlex 3.0 databases revealed an enrichment of database membership by 52.2- and 86.1-fold, respectively compared to the non-correlated pairs (**Supplementary Figure 1G-H**). Similarly, the higher positive correlation coefficients were associated with increased STRINGdb linkscores (**Supplementary Figure 1I**). These results indicate that highly correlated protein pairs discovered in this workflow are genuine associations, based on previously reported protein-protein interactions. Consistent with previous co-regulation network analysis, top CORUM protein complexes identified included mitochondrial complex I, ribosome, proteasome, and spliceosome. Furthermore, it is expected that co-regulated proteins have a larger proportion of overlapping annotations. Indeed, among the correlated protein pairs almost 40% share a subcellular localization compared to less than 10% for the non-correlated pairs (**Supplementary Figure 2I**).

pQTL and lipid-QTL summary

A total of 200,628 SNPs were associated to the abundance of 978 proteins of which 90% were associated to 789 proteins via *cis*-pQTLs (+/- 10 mb, $p < 1e^{-4}$) and 10% were associated to 436 proteins via *trans*-pQTLs ($p < 1e^{-7}$) (**Supplementary Figure 1K**). Furthermore, 1,336 SNPs were identified within 336 genes and were also associated to the expression of these corresponding proteins via *cis*-pQTLs. Although most variants are modifier-impact (>98%), small numbers of low- (1,982, 1%), moderate- (913, 0.4%), and high-impact (35, 0.02%) SNPs were found (**Supplementary Figure 1L**). Annotating these SNPs with Ensembl variant effects (**Supplementary Figure 1M**) revealed that most SNPs are intron (31%) or intergenic (30%) variants. A total of 35 high-impact SNPs were associated to protein abundance via variants in stop codons or splicing sites (**Supplementary Figure 1N**). We also performed variant effect analysis on 1,808 SNPs that were associated to 89 lipid species and 17 lipid classes. The dataset contained 1,338 associations with lipid classes and 6,694 associations with individual liver lipids.

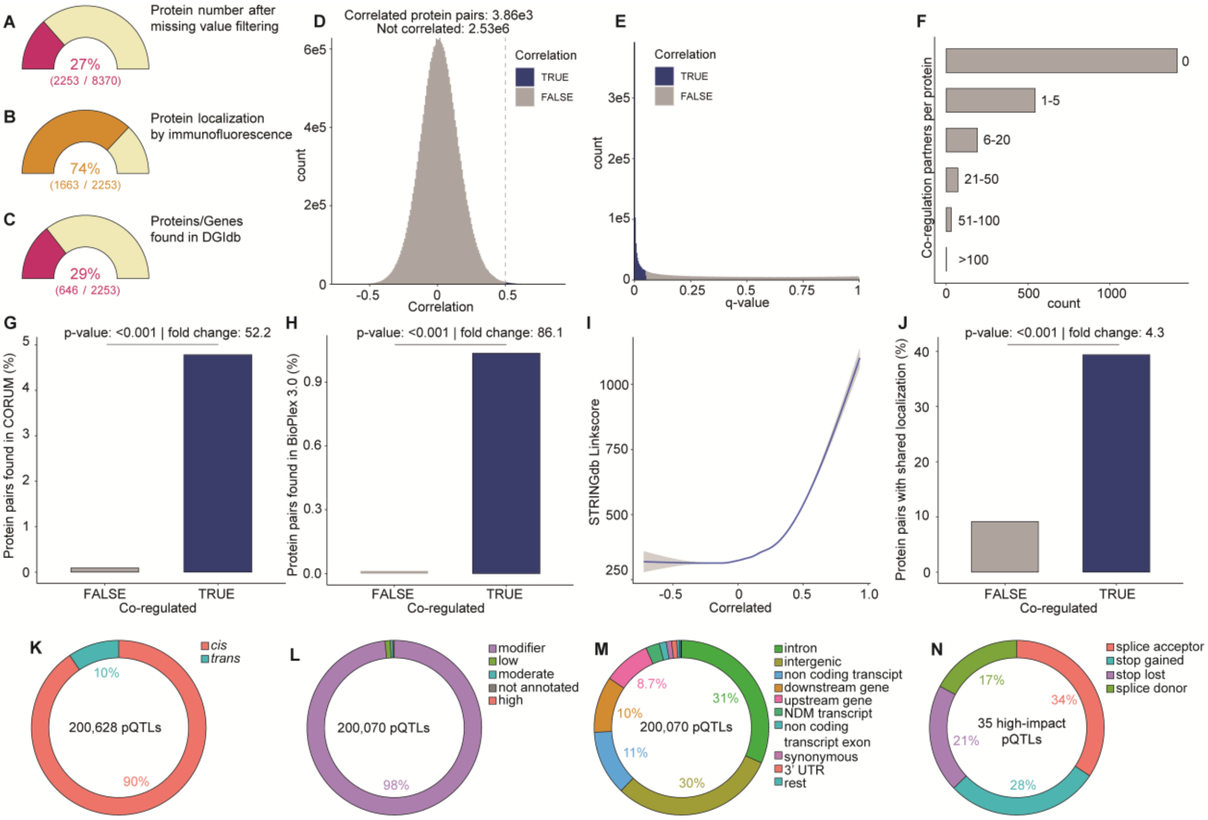

Supplementary Figure 1. *CoffeeProt* pre-processing & correlation summary. (A) Percentage of proteins filtered by user-specified missing value cut-off. (B) Percentage of proteins annotated with Cell Atlas protein localization as determined by immunofluorescent staining. (C) Percentage of proteins present in the Drug-Gene Interaction Database. The number of co-regulated protein pairs after filtering for user-specified correlation (D) or q-value (E) cut-offs. The number of protein pairs that

meets the criteria is displayed in both plots. The number of co-regulation partners per proteins (F) has been determined based on the user-specified correlation cut-off. The data is binned to group proteins with 0, 1-5, 6-20, 21-50, 51-100 and >100 partners. Enrichment of previously reported protein-protein interactions as reported in the CORUM (G), BioPlex 3.0 (H) and STRING (I) databases. Enrichment of protein pair co-localization (J) was determined based on Cell Atlas localization determined by immunofluorescent staining. A Chi-squared test was performed to determine the significance of enrichments. pQTL annotation distributions by proxy (K), variant impact (L) and variant effect (M, N).

CASE STUDY: Genomic atlas of the human plasma proteome

In this study, SOMAscan-based proteomics was used to quantify over 3,200 proteins in the plasma of 3,301 healthy participants and integrated with genomic data via pQTL analysis (2). Access to the proteomics and pQTL data was provided upon request through the European Genotype Archive (accession number EGAS00001002555). The data was simply pre-processed to filter columns and uploaded directly to *CoffeeProt* for further analysis. The proteomics data was filtered to exclude proteins with more than 20% missing values amongst the samples, leaving a dataset containing 3,257 proteins (**Supplementary Figure 2A**). Among these proteins, 1,863 (57%) were annotated by protein localization as determined using immunofluorescent staining as reported in the Human Protein Atlas (3) (**Supplementary Figure 2B**) and 1153 (35%) were present in the Drug-Gene Interaction Database (**Supplementary Figure 2C**).

Protein correlation summary

Like the first case study, we performed protein-protein correlation using the biweight midcorrelation (bicor), followed by the Benjamini-Hochberg procedure. We considered proteins with a bicor coefficient > 0.5 and q-value < 0.05 to be correlated. This correlation coefficient cut-off led to 1% ($5.53e^4$ out of $5.48e^6$) of the protein-pairs to be defined as correlated (**Supplementary Figure 2D-E**). Using these cut-offs, most proteins have no or a small number (1-5) of co-regulation partners (**Supplementary Figure 2F**). A smaller group of proteins have many co-regulation partners, indicating proteins that are likely central in one or multiple protein complexes.

Protein correlation database enrichment

Analyzing the fraction of previously reported protein pairs in the correlation matrix based on the CORUM and BioPlex 3.0 databases revealed an enrichment of database membership by 8.2- and 9.4-fold, respectively compared to the non-correlated pairs (**Supplementary Figure 2G-H**). However, it should be noted that only a small fraction of the quantified plasma proteome was mapped to these databases presumably because protein-protein interaction analysis has previously focused on intracellular proteins. Similarly, the higher positive correlation coefficients were associated with increased STRINGdb linkscores (**Supplementary Figure 2I**). These results indicate that despite the

low annotation of plasma protein complexes, highly correlated protein pairs discovered in this workflow are genuine associations, based on previously reported protein-protein interactions.

pQTL summary

The pQTL data as processed by Sun *et al.* were filtered by p-value ($p < 1e^{-4}$) and all resulting tables were merged, resulting in a table of ~5,000,000 pQTLs. The SOMAmer identifiers in the QTL data were annotated by mapping to their respective gene names and locations (chromosome and bp) on the GRCh37/hg19 reference genome. UniProt identifiers were converted to gene names using the AnnotationDbi and org.Hs.eg.db R packages. GENCODE human release 19 (CRCh37.p13) was used to retrieve genomic locations for all measured proteins. We defined SNPs located < 1MB from the start of the target gene as *cis*-pQTLs, whereas all other SNPs are defined as *trans*-pQTLs. The resulting pQTL file was uploaded to *CoffeeProt* for further filtering, analysis and visualization. Both *cis*- and *trans*-pQTLs were filtered to $p < 1e^{-11}$ consistent with the original publication resulting in the identification of 324,537 SNPs associated to the abundance of 1,409 proteins of which 46% were associated to 530 proteins via *cis*-pQTLs and 53% were associated to 1,066 proteins via *trans*-pQTLs (**Supplementary Figure 2J**). Although most variants were modifier-impact (94%), small numbers of low- (2215, 0.6%), moderate- (3027, 0.9%), and high-impact (160, 0.008%) SNPs were found (**Supplementary Figure 2K**). The majority of SNPs annotated with the Ensembl variant effects were intergenic (35%), intron (33%), or non-coding transcript variants (17%) (**Supplementary Figure 2L**). The high-impact variants were categorized as stop gained- (52), frameshift- (40), splice donor- (30), splice acceptor- (30), start lost- (4) or stop lost (4) variants (**Supplementary Figure 2M**).

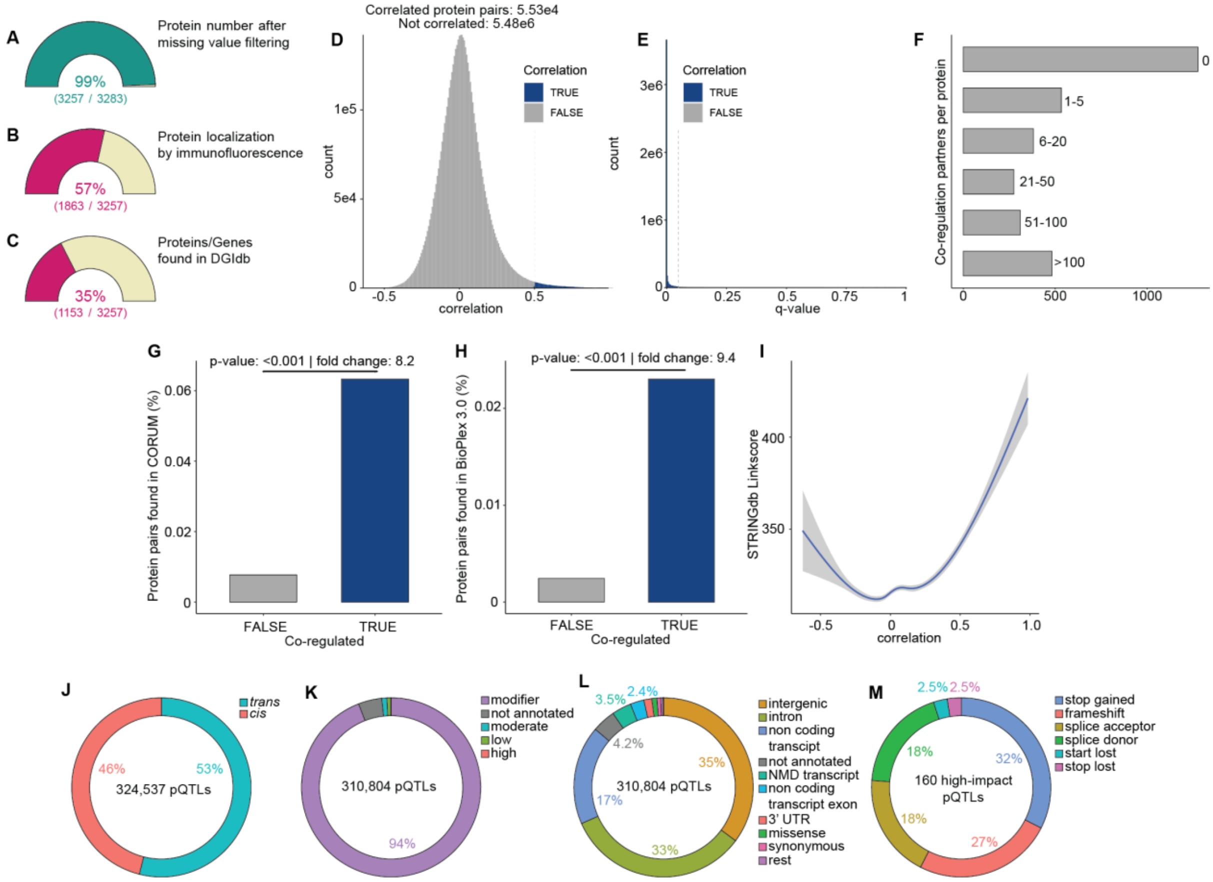

Supplementary Figure 2. *CoffeeProt* pre-processing & correlation summary. (A) Percentage of proteins filtered by user-specified missing value cut-off. (B) Percentage of proteins annotated with Cell Atlas protein localization as determined by immunofluorescent staining. (C) Percentage of proteins present in the Drug-Gene Interaction Database. The number of co-regulated protein pairs after filtering for user-specified correlation (D) or q-value (E) cut-offs. The number of protein pairs that meets the criteria is displayed in both plots. The number of co-regulation partners per proteins (F) has been determined based on the user-specified correlation cut-off. The data is binned to group proteins with 0, 1-5, 6-20, 21-50, 51-100 and >100 partners. Enrichment of previously reported protein-protein interactions as reported in the CORUM (G), BioPlex 3.0 (H) and STRING (I) databases. A Chi-squared test was performed to determine the significance of enrichments. pQTL and molQTL annotation distributions by proxy (J), variant impact (K) and variant effect (L, M).

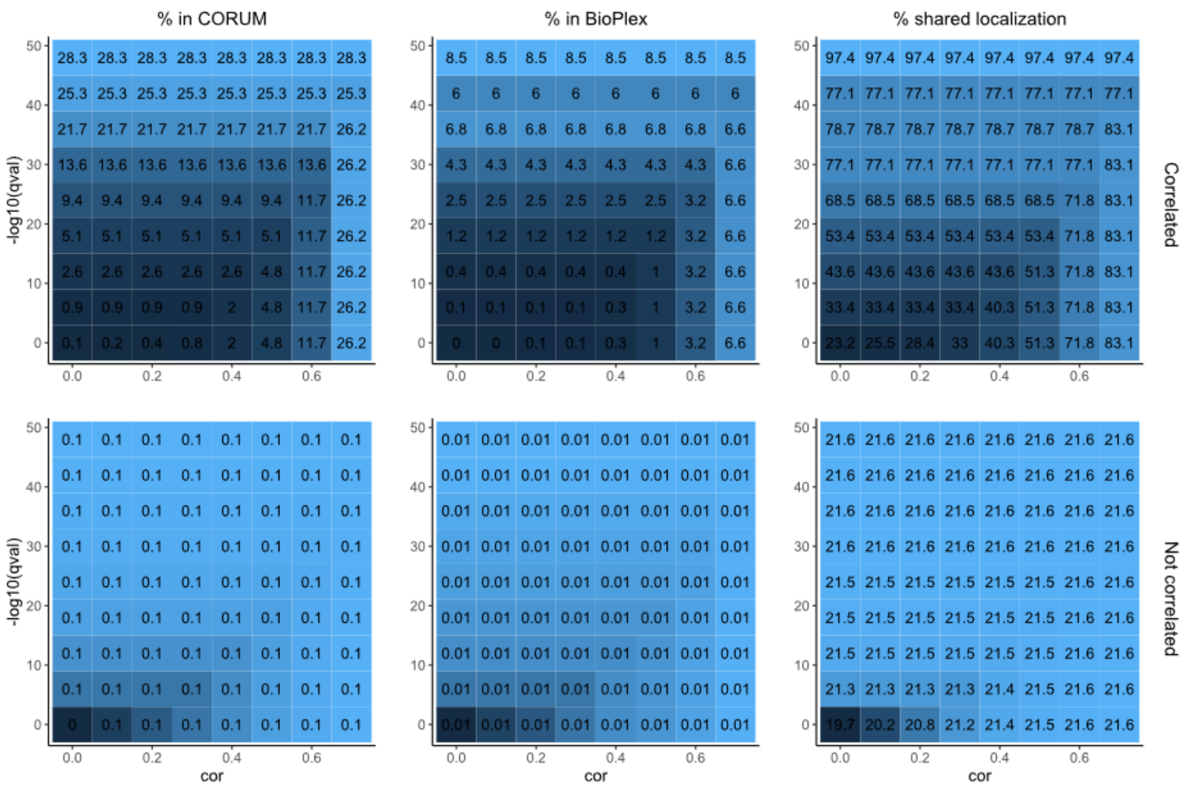

Supplementary Figure 3. Sensitivity analysis of the relationship between database enrichment input parameters and outcomes. The fraction of protein pairs found in protein-protein interaction databases, or with shared localizations, was determined for correlated and uncorrelated pairs. Testing over 70 combinations of input parameters allows users to select suitable cut-off parameters for subsequent analyses.

References

1. Parker, B.L., Calkin, A.C., Seldin, M.M., Keating, M.F., Tarling, E.J., Yang, P., Moody, S.C., Liu, Y., Zerenturk, E.J., Needham, E.J. *et al.* (2019) An integrative systems genetic analysis of mammalian lipid metabolism. *Nature*, **567**, 187-193.
2. Sun, B.B., Maranville, J.C., Peters, J.E., Stacey, D., Staley, J.R., Blackshaw, J., Burgess, S., Jiang, T., Paige, E., Surendran, P. *et al.* (2018) Genomic atlas of the human plasma proteome. *Nature*, **558**, 73-79.
3. Thul, P.J., Akesson, L., Wiking, M., Mahdessian, D., Geladaki, A., Ait Blal, H., Alm, T., Asplund, A., Bjork, L., Breckels, L.M. *et al.* (2017) A subcellular map of the human proteome. *Science*, **356**.
